# Student wellbeing supports in Australian and New Zealand medical schools: A post-guidance snapshot from a 14-institution survey

**DOI:** 10.1177/00048674261455737

**Published:** 2026-06-23

**Authors:** Andri Burger, Chanaka Wijeratne, Minh Thu Nguyen, Megan Kalucy, Kimberlie Dean

**Affiliations:** 1 Discipline of Psychiatry and Mental Health, School of Clinical Medicine, UNSW, Sydney, NSW, Australia

## Introduction

Medical students experience higher rates of psychological distress and a range of mental health problems compared with population peers, including elevated depression and suicidal ideation, with implications for learning and patient safety (Moir et al., 2023; Rotenstein et al., 2016). In Australia and New Zealand, recent guidance emphasises safe learning environments and wellbeing-oriented curricula, including the Medical Deans of Australia and New Zealand (MDANZ) consensus statement, the Every Doctor, Every Setting framework and revised Australian Medical Council (AMC) accreditation standards (Australian Medical Council Limited, 2023; Everymind, 2019; Kemp et al., 2019). We conducted a national survey to evaluate the alignment of current student wellbeing supports and policies with established industry standards (*Every Doctor, Every Setting* and *MDANZ Consensus Statement*) and to identify barriers to their implementation across the sector.

## Methods

We invited all 24 AMC-accredited medical schools in Australia and New Zealand to complete an online survey between February and May 2024. Recruitment occurred via MDANZ and MHELANZ Basecamp portals and direct emails, with one initial invitation and two follow-ups. Participants were recruited via these professional networks and direct email, targeting staff with roles in student wellbeing or medical education; however, responses reflect individual institutional perspectives rather than formally endorsed organisational positions. Responses were completed via Qualtrics or, for one school, via teleconference, with responses recorded into Qualtrics by a researcher.

Sixteen responses were received from 14 schools (58% of all schools). Duplicate responses (two schools) were resolved by retaining the more complete entry. Final respondents comprised five educators (e.g. professors, course coordinators, heads of discipline) and nine administrators (e.g. deans of student affairs, wellbeing liaison officers, student support officers, mental health convenors), all with roles relevant to student wellbeing or medical education. The survey instrument was developed by the research team and mapped to the pillars of the *Every Doctor, Every Setting* framework and *MDANZ Consensus Statement* topic areas. Items were structured to evaluate institutional alignment with AMC 2023 standards. Prior to distribution, the instrument was reviewed by the research team for face validity and clarity. Domains included student mental health support and services; curriculum and learning environment; staff development; guideline awareness and integration and barriers. Items comprised binary and multiple-choice questions with short free-text prompts. Quantitative data were summarised descriptively. Free-text responses were inductively coded by one reviewer and checked by a second; given the brief format and small sample, analysis was exploratory and used to illustrate key themes within the descriptive results. Missing responses were treated listwise; percentages reflect valid responses per item.

## Results

### Participants

Fourteen schools responded (13 Australia, 1 New Zealand). Respondents were institutional staff responsible for student wellbeing, education or administration.

### Supports and services

Most schools reported internal wellbeing services (11/14, 78%), primarily psychologists, counsellors and general practitioners; nearly all facilitated access to external services (12/14, 86%). Scope and models varied by institution (Table 1). Several respondents noted challenges sustaining equitable access for international students.

**Table 1.** Student wellbeing supports, policies and training reported by responding medical schools (*n* = 14).

Domain	Item	n (%)
Health services	Internal wellbeing services Facilitation of external access	11 (78) 12 (86)
Institutional	Dedicated wellbeing committee Student stakeholder engagement structures	8 (57) 12 (86)
Policies	Separation of academic progression and wellbeing support	10 (72)
Learning environment	Wellbeing embedded in curriculum and/or assessment	12 (86)
Staff training	Managing distressed students Giving constructive feedback Difficult conversations/disclosures	7 (50) 9 (64) 8 (57)

Denominator is 14 unless specified; where an item was left blank, percentages reflect valid responses. ‘Separation’ refers to firewalls between support services and assessment/progression decisions. ‘Student stakeholder engagement structures’ refers to formal forums (e.g. committees, co-design groups) where students contribute to wellbeing policy and practice.

### Curriculum and learning environment

Twelve schools (12/14, 86%) reported embedding wellbeing within teaching and/or assessment, including resilience-building, stigma reduction and promotion of healthy behaviours. One respondent described ‘weekly small-group professional practice sessions from year 1 to discuss sensitive issues’. A safe culture was widely reported, though local approaches differed.

### Guideline awareness and integration

Awareness of national frameworks was common (11/14, 78%). No school provided formal evidence of full integration; seven (7/14, 50%) reported active processes to integrate recommendations.

### Staff training and policies

Most schools reported providing some wellbeing-related staff training; topic-specific coverage varied: training on managing distressed students (7/14, 50%), giving constructive feedback (9/14, 64%) and handling difficult conversations/disclosures (8/14, 57%). Separation of academic progression and wellbeing support policies (‘firewalls’) were present in 10/14 (72%).

### Barriers

Frequently cited barriers included funding constraints, limited staff training coverage and competing institutional priorities, with respondents noting, e.g., ‘Not enough staff. Staff to student ratio is imbalanced; wellbeing is only one part of staff roles’. Several respondents also highlighted inequities for high-risk groups, particularly international students, and the need for structured follow-up processes.

## Discussion

This study provides a post-guidance benchmark for medical student wellbeing supports, revealing that while initiatives are widely reported to be embedded in curricula, substantial gaps remain in aligning local practice with national framework recommendations. Findings reflect partial but uneven progress towards sector guidance issued since 2019 and revised accreditation standards in 2023. However, supports varied in scope, formal integration of national frameworks was incomplete and training coverage across key topics was uneven. Commonly reported barriers – funding constraints, training capacity and competing priorities – mirror international experience with wellbeing initiatives in medical education and underscore the need for system-level approaches (Gulliver et al., 2010; Klein and McCarthy, 2022; Slavin et al., 2014). This post-guidance snapshot provides baseline benchmarking following MDANZ and AMC developments. We suggest three pragmatic, accreditation-aligned targets to promote consistent implementation:

1. Define minimum service standards with equitable access (including international students), such as response times, after-hours pathways and clear referral options to on-site or affiliated health services.
2. Specify core educator competencies (recognition, response, referral) with coverage targets for academic and clinical supervisors, supported by brief, scalable training (e.g. short online modules or case-based workshops).
3. Embed periodic benchmarking indicators within accreditation cycles (e.g. presence and operation of separation-of-care policies, staff training coverage and evidence of wellbeing integration within assessment practices). While our benchmarking focuses on institutional reports, we acknowledge that meaningful progress requires the integration of student voices to ensure that reported structures align with the lived experience of student wellbeing.

Limitations include a modest response rate (58%), predominance of Australian responses with minimal representation from New Zealand (*n* = 1) and reliance on staff-reported institutional perspectives, which may not reflect whole-of-institution practices or student experiences. We were unable to assess systematic differences between responding and non-responding schools; it is possible that institutions with more developed wellbeing initiatives were more likely to participate. Findings should therefore be interpreted as descriptive of responding institutions rather than all medical schools. Australian and New Zealand medical schools report substantive efforts to support student wellbeing, yet gaps in equity, integration and sustainability remain. Strengthening accountability through accreditation, resourcing minimum standards and expanding staff training coverage may accelerate consistent implementation across the sector.
